# Extracellular vesicles released by in vitro plant cell cultures: emerging systems for vesicle-mediated communication and biomedical translation

**DOI:** 10.1007/s00299-026-03983-7

**Published:** 2026-09-21

**Authors:** Francesca Cristiana Piritore, Arianna Minoia, João Pessoa, Maria Grazia Romanelli, Luca Dalle Carbonare, Maria Teresa Valenti

**Affiliations:** 1 Department of Neurosciences, Biomedicine and Movement Sciences, University of Verona, Verona, Italy; 2 Department of Engineering for Innovation Medicine, University of Verona, Verona, Italy; 3 Azienda Ospedaliera Integrata Verona, Verona, Italy; 4 CICECO - Aveiro Institute of Materials, LAQV-REQUIMTE, Department of Chemistry, University of Aveiro, Aveiro, Portugal

**Keywords:** Plant extracellular vesicles, Intercellular communication, Cellular signaling, Bioactive cargo, Extracellular vesicle biogenesis

## Abstract

**Key message:**

This article summarizes how extracellular vesicles are emerging as important means through which cells communicate with each other in plants and their potential medical applications.

**Abstract:**

Extracellular vesicles are emerging as important mediators of intercellular communication in plants, with roles in defense responses, stress adaptation, and cross-kingdom molecular signaling. Although plant-derived extracellular vesicles have attracted increasing interest, most studies have focused on vesicles isolated from whole tissues or edible plant materials, whereas extracellular vesicles released by in vitro plant callus and suspension cell cultures remain comparatively underexplored. This review discusses plant cell cultures as controllable and potentially scalable experimental systems for investigating extracellular vesicle biogenesis, molecular cargo, isolation, characterization, and biological activity. Particular attention is given to the distinction between bona fide extracellular vesicles secreted by living plant cells and heterogeneous nanovesicle preparations generated through tissue disruption, a key issue for reproducibility and interpretation in plant EV research. We also summarize current evidence on vesicle-mediated communication, cargo modulation, engineering strategies, and potential biomedical applications. Finally, we highlight major methodological gaps, including the lack of plant-specific markers, standardized isolation workflows, quality control criteria, and harmonized reporting practices. By integrating plant cell biology, extracellular vesicle research, and plant biotechnology, in vitro plant cell cultures may provide useful model systems for studying vesicle-mediated communication and for developing biologically produced vesicles with more defined functional properties.

## Introduction

Extracellular vesicles (EVs) are membrane-bound nanoparticles released into the extracellular milieu, typically ranging from 30 to 300 nm in diameter (Gill et al. 2019; Pavlova 2025). The following discussion specifically concerns EVs produced by mammalian cells. In mammalian systems, EVs—including exosomes, microvesicles, and apoptotic bodies—are now widely recognized as central mediators of intercellular communication, immune regulation, and disease progression, and are actively investigated as diagnostic biomarkers and therapeutic tools (Kumar et al. 2024). Among these vesicle populations, exosomes exhibit features that make them particularly attractive as drug delivery vehicles. Their membrane composition preserves surface proteins involved in cell recognition and adhesion, enabling efficient interaction with recipient cells and facilitating targeted delivery of therapeutic cargos (Ren et al. 2016). Furthermore, exosome-based platforms allow the manipulation of parental cells to display specific targeting ligands or to selectively enrich vesicles with bioactive molecules, broadening their applicability across a wide range of pathological conditions, including cancer, infectious diseases, cardiovascular disorders, and neurodegenerative diseases (Yang et al. 2022). By combining the advantages of synthetic nanocarriers with those of cell-mediated delivery systems, exosomes represent a versatile and efficient strategy for therapeutic cargo delivery. Compared with conventional nanotechnologies, they offer improved biocompatibility, enhanced targeting capacity, prolonged circulation, and reduced immunogenicity (Martínez-Santillán and González-Valdez 2023).

In contrast, vesicle-mediated secretion in plants remained largely underexplored for decades and was often regarded as a secondary consequence of cell wall remodeling or secretion (Rizzo et al. 2021). This perspective has changed substantially over the past fifteen years, following seminal studies demonstrating that plant cells actively release EVs enriched in specific proteins, lipids, and RNA species (Woith et al. 2019; Rizzo et al. 2021). In plants, several intracellular compartments have been proposed as potential sources of EV biogenesis, including the exocyst-positive organelle (EXPO), multivesicular bodies (MVBs), vacuoles, and autophagosomes (Wang et al. 2010; Robinson et al. 2016). EV-mediated information transfer has been shown to contribute to plant defense mechanisms, stress responses, and inter-organism communication (De Meo et al. 2025; Srivastava et al. 2025). Notably, EV secretion has been documented, not only in intact plant tissues, but also in plant cells maintained in vitro, including callus and suspension cultures, highlighting EV release as an intrinsic cellular process rather than a tissue-dependent phenomenon (Yugay et al. 2023; Kocholatá et al. 2024; Kırbaş et al. 2025). Thus, plant-derived extracellular vesicles (PDEVs), often referred to as plant-derived exosomes (PDEs), are now recognized as active mediators of cell-to-cell communication that contribute to the regulation of physiological and pathological processes through defined biological activities (Kırbaş et al. 2025; Yazdanpanah et al. 2025). Although their therapeutic potential has received limited attention until recently, accumulating evidence indicates that PDEVs function as carriers of signaling molecules capable of modulating the cellular microenvironment in mammalian experimental systems (Yazdanpanah et al. 2025). This functional versatility underpins their emerging relevance in diverse applications, including antioxidant and anti-inflammatory responses, immune modulation, inhibition of tumor cell growth, and promotion of tissue repair and regeneration. In parallel, PDEVs have gained increasing interest as natural nanocarriers for drug delivery, as they can enhance the stability, bioavailability, and targeted transport of therapeutic agents to specific cells or tissues. Collectively, these properties position PDEVs as promising tools for targeted drug delivery and the development of innovative therapeutic strategies. These biomedical applications, which have mainly been investigated in mammalian cells and animal models, should be distinguished from the physiological functions of EVs in plants. A major challenge in the field of plant EVs is the lack of standardized nomenclature (Pinedo et al. 2021; Rodríguez de Lope et al. 2024). Unlike mammalian systems, where vesicle subclasses are operationally defined (e.g., exosomes as endosome-derived vesicles), plant EVs lack definitive molecular markers that unambiguously distinguish their biogenetic pathways (Rodríguez de Lope et al. 2024). Consequently, terms such as “extracellular vesicles,” “exosome-like vesicles,” and “plant-derived nanovesicles” are used interchangeably. Most authors currently avoid the strict use of the term “exosome,” as direct evidence for multivesicular body–plasma membrane fusion analogous to mammalian exosome release remains incomplete in plants (An et al. 2007; Ratajczak and Ratajczak 2020; Dad et al. 2021). In line with recent consensus, this review adopts the umbrella term “extracellular vesicles (EVs)” while acknowledging that multiple vesicle subpopulations likely coexist. Given the growing interest in plant EVs, this review focuses on extracellular vesicles released by plant cells cultured in vitro, with particular emphasis on suspension cultures as controlled and scalable platforms for EV production. We summarize current knowledge on EV biogenesis, isolation and characterization strategies, molecular cargo, and biological functions, and discuss their emerging biomedical applications, particularly as natural nanocarriers for drug delivery. Finally, we highlight the major methodological challenges, current knowledge gaps, and future perspectives for the development and standardization of plant EV research, in line with the rapidly evolving research landscape, principal hotspots and emerging trends identified by recent bibliometric mapping of the field (Shu et al. 2026).

## Biological functions and experimental models of plant extracellular vesicles

Plant EVs (PDEVs) are also known as functional components of the plant intercellular communication system and as simple products of specialized secretion (Fu et al. 2025). Experimental studies based on apoplast isolation, vegetal tissues, and cellular culture have shown that these EVs include selective molecular cargos consisting of proteins involved in defense, metabolic enzymes, structural lipids, and small RNA regulators (Rutter and Innes 2017; Calzoni et al. 2024). The content of EVs is dynamic and reflects the cell's physical state, suggesting regulated processes of biogenesis and chemistry (Jin et al. 2022). Proteomic analyses have revealed enrichment of proteins involved in immunological responses, cellular remodeling, and oxidative stress management, suggesting that EVs contribute to the fine regulation of cellular responses. In addition, comparative proteomic analyses of plant extracellular vesicles (PDEVs) and other plant-derived vesicles have identified conserved and unique protein signatures associated with bona fide plant EVs (Bifolco et al. 2026). Experimental evidence has also shown that PDEVs isolated during systemic acquired resistance (SAR) can amplify systemic immune signaling and respond differentially to biotic stress (Wang et al. 2025a, b). Furthermore, EVs can be taken up by plant cells, indicating a direct role in the transfer of biochemical signals between cells (Koch et al. 2024; Wang et al. 2025a, b). In this context, EVs may act as signal communicators by transferring cell type-specific molecular cargo between plant cells. Because different cell types can produce distinct EV populations and respond differently to stress, EV-mediated signaling may contribute to coordinating these responses at the tissue and whole-plant levels.

### Plant–plant and plant–pathogen communication

**Fig. 1:**
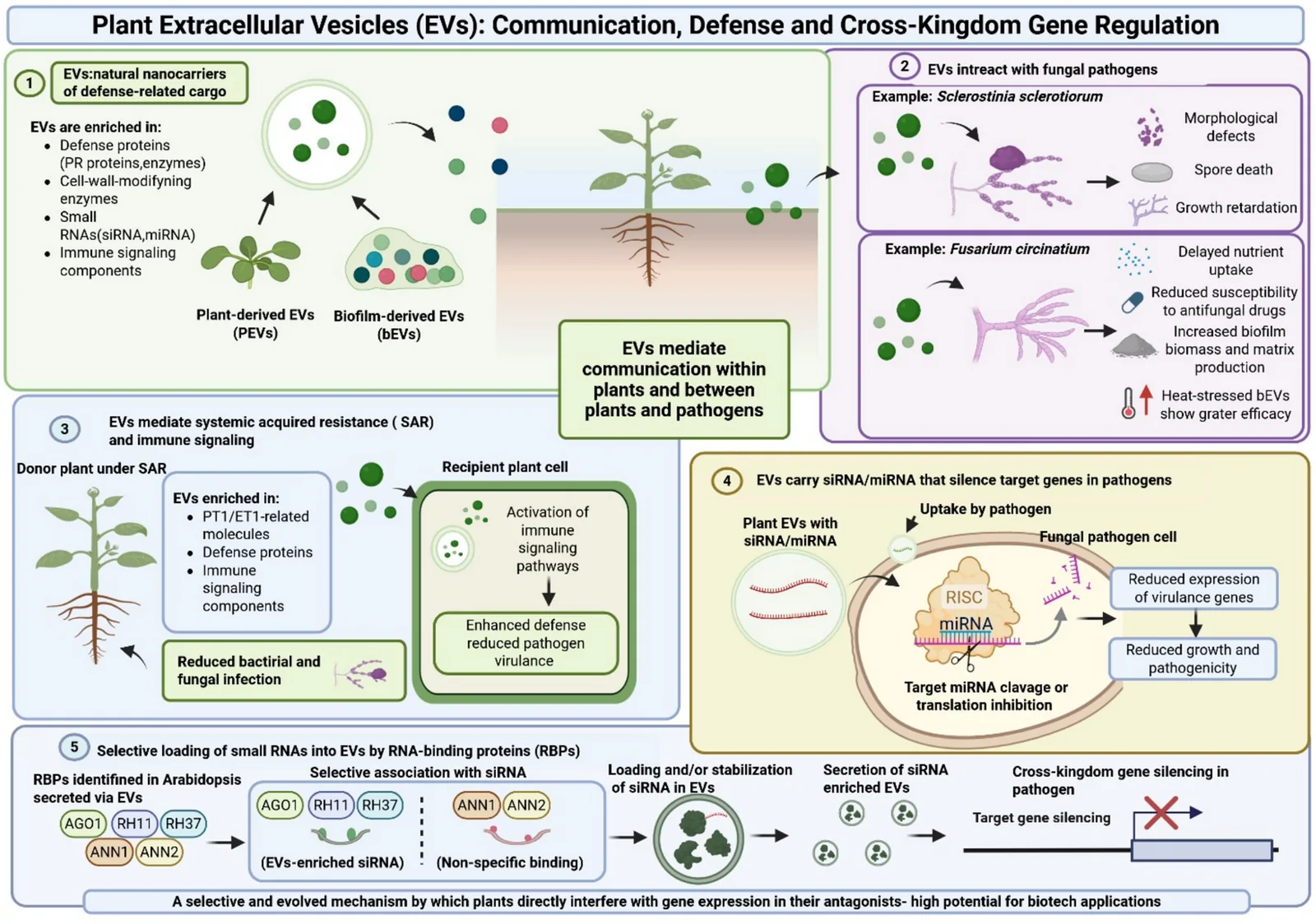
Schematic overview of the role of plant extracellular vesicles (PDEVs) in plant defense and communication. (1) Plant-derived EVs and biofilm-derived EVs (bEVs) carry defense proteins, immune signaling molecules, and small RNAs (siRNAs and miRNAs), supporting communication within plants and between plants and pathogens. (2) PDEVs interact with fungal pathogens, such as *Sclerotinia sclerotiorum* and *Fusarium circinatum*, reducing pathogen growth and virulence through multiple mechanisms. (3) During systemic acquired resistance (SAR), EVs released by donor plants are taken up by recipient plant cells, where they activate immune signaling pathways and enhance defense responses. (4) EV-associated siRNAs and miRNAs can be transferred to fungal pathogens, where they silence target genes involved in pathogenicity, thereby reducing pathogen virulence. (5) The selective loading of small RNAs into EVs is mediated by RNA-binding proteins, including AGO1, RH11, RH37, ANN1, and ANN2, which contribute to sRNA packaging, stabilization, and cross-kingdom gene silencing. Together, these mechanisms highlight the importance of PDEVs in plant immunity and intercellular as well as cross-kingdom communication. This schematic represents an idealized overview, as different plant cell types may produce distinct EV populations containing specific signaling molecules. This image was created with BioRender (https://biorender.com/)

PDEVs are important for communication among plants and between plants and pathogens because they carry defense-related chemicals and regulatory molecules capable of activating signaling pathways in recipient cells, as evidenced by an increasing body of experimental data (Cai et al. 2018; Wang et al. 2025a, b). One of the earliest functional demonstrations of these processes was the characterization of sunflower EVs and their potential to interact with fungus cells. These nanovesicles are rich in defense proteins and enzymes that modify cell walls (Regente et al. 2017). Moreover, biofilm-derived EVs (bEVs) applied to fungal cells demonstrated their potential to support conidial viability and germination (Fig. 1). It was confirmed that the phytopathogenic fungus *Sclerotinia sclerotiorum* absorbed membrane-labeled EVs. In addition, spores treated with plant EVs showed morphological defects, death, and growth retardation, establishing the presence of these EVs and showing that they can bind and kill fungal cells (Regente et al. 2017). Again, *Fusarium circinatum* treated with bEVs showed delayed uptake of nutrients and reduced susceptibility to antimicrobial drugs. Moreover, the external application of bEVs to mono- and polymicrobial biofilms significantly enhanced biomass and matrix production. Notably, EVs from heat-stressed biofilms exhibited greater efficacy in promoting biomass production and increasing resistance to antifungal agents (Ratsoma et al. 2025). Evidence from Arabidopsis showed that plant EVs contribute to immune signaling between plant cells during SAR (Wang et al. 2025a, b). In fact, EVs derived from plants undergoing SAR are rich in molecules associated with pattern-triggered and effector-triggered immunity, defense proteins, and immune signaling components (Wang et al. 2025a, b). The recipient plant cells take up EVs, whose molecular cargo can activate immune signaling pathways. As a result, the virulence of bacterial and fungal pathogens is reduced, as demonstrated by both in planta infection assays and in vitro neutralization tests, both consistent with the observed functional outcomes (Wang et al. 2025a, b). Further complexity is highlighted by experimental evidence demonstrating that plant EVs carry small interfering RNAs (siRNAs) and micro RNAs (miRNAs) capable of silencing target genes in interacting pathogens, thereby providing direct evidence for EV-mediated cross-kingdom gene regulation**.** He et al. discovered several RNA-binding proteins (RBPs) in *Arabidopsis* that are secreted via exosome-like EVs, including argonaute 1 (AGO1), RNA helicases (RHs), and annexins (ANN) (He et al. 2021). AGO1, RH11, and RH37 were found to selectively associate with EV-enriched small RNAs, whereas ANN1 and ANN2 showed non-specific sRNA binding. Accordingly, the corresponding mutants (*ago1, rh11 rh37, and ann1 ann2*) displayed reduced sRNA secretion in EVs, suggesting that these RNA-binding proteins are crucial for sRNA loading and/or stabilization within EVs (He et al. 2021). Thus, it is a very selective and evolved mechanism by which plants can directly interfere with gene expression in their antagonist, showing an extremely high potential for biotechnological applications. Although RNA-binding proteins contribute to selective sRNA loading, the relationship between cargo selection and specific EV biogenesis pathways remains poorly defined. ESCRT-dependent formation of multivesicular bodies may promote the compartmentalization of particular RNA–protein complexes into EV subpopulations, whereas EXPO-, autophagy- or vacuole-associated routes may provide non-canonical secretion mechanisms. It remains unclear whether these pathways recruit distinct RNA-binding proteins or package specific sRNA subsets for cross-kingdom delivery. Genetic perturbation of individual biogenesis components, combined with EV subpopulation separation and parallel RBP and sRNA profiling, will be required to establish a causal relationship between secretion pathway and selective RNA cargo (Wang et al. 2010; Robinson et al. 2016; He et al. 2021; Yugay et al. 2023).

Plant cell cultures are relevant for EV research because they allow controlled investigation of how oxidative, mechanical, osmotic or nutritional treatments affect cellular metabolism and EV secretion. However, callus and suspension cultures are relatively homogeneous and lack the tissue organization, cell-type diversity, endogenous hormonal gradients and epigenetic heterogeneity of intact plants. Therefore, they provide information on cell-autonomous responses under in vitro conditions but cannot reproduce the coordinated stress response of the whole plant (Woith et al. 2021). EVs generated under these in vitro conditions are enriched in stress-related proteins and regulatory RNAs, suggesting their involvement in cell-autonomous defense or acclimation responses rather than whole-plant adaptation (Regente et al. 2017; Yugay et al. 2023). This distinction is important because suspension cultures are commonly maintained with exogenous growth regulators, whereas stress adaptation in planta depends on endogenous growth-regulator gradients and coordinated responses among differentiated cell types.

### In vitro plant cell cultures as platforms for EV production

**Fig. 2:**
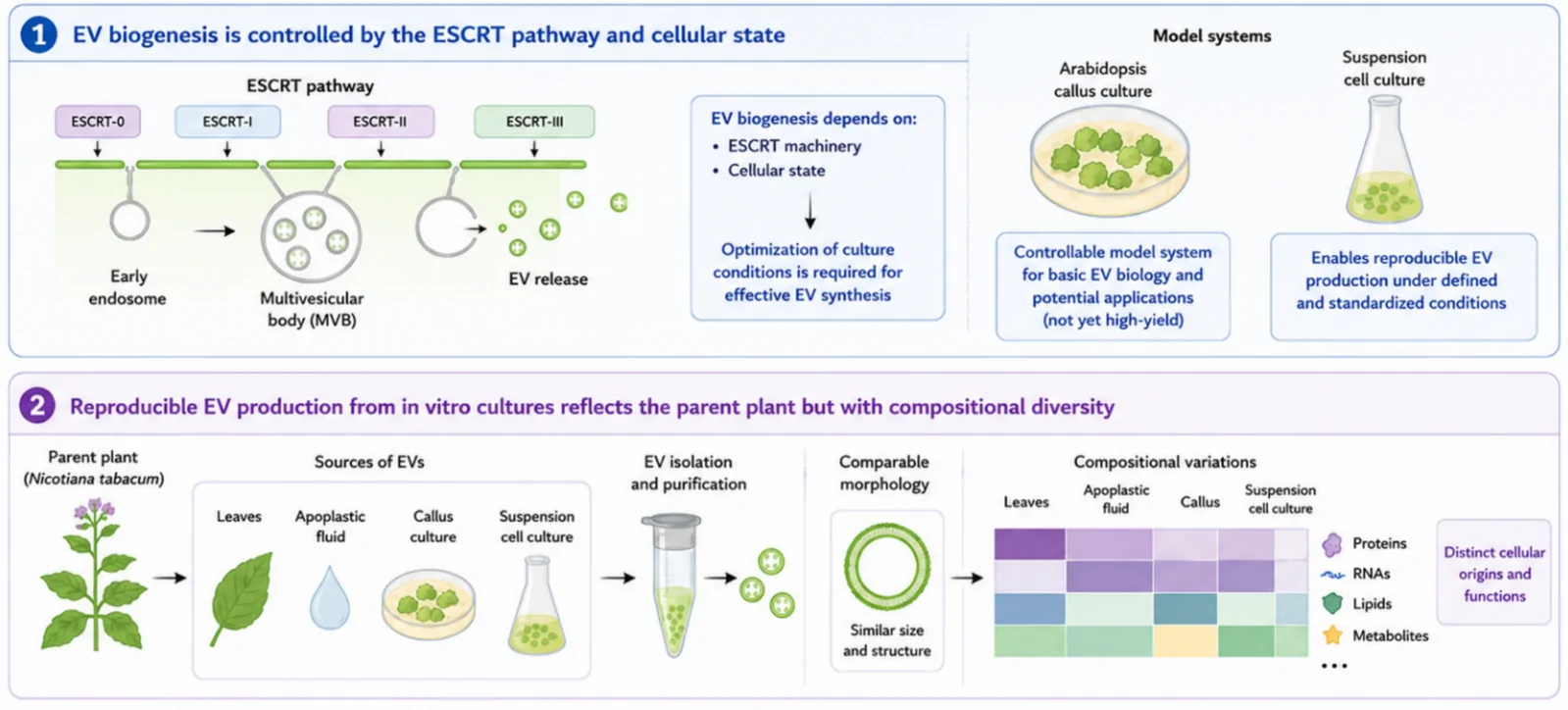
Plant cell cultures as controllable systems for extracellular vesicle (EV) production. (1) EV biogenesis is regulated by the ESCRT pathway and cellular state, highlighting the importance of optimizing culture conditions for efficient EV production. Arabidopsis callus and suspension cell cultures provide controllable experimental systems for studying EV biology under defined conditions. (2) EVs can be reproducibly isolated from different plant tissues and in vitro cultures, including leaves, apoplastic fluid, callus, and suspension cell cultures. Although EVs display comparable morphology, differences in their molecular composition reflect distinct cellular origins and biological functions. This idealized model highlights the experimental advantages of plant cell cultures but does not reproduce the cellular diversity, tissue organization or endogenous signaling gradients of intact plants. *This image was created with BioRender (*https://biorender.com/*)*

Yugay et al. showed that while plant cell cultures can generate specific EVs whose cargo reflects important characteristics of the parent plant, EV biogenesis depends on the control of the endosomal-sorting complex required for transport (ESCRT) pathway and cellular state (Yugay et al. 2023). This finding highlights the need to optimize culture conditions for effective EV synthesis while positioning *Arabidopsis* callus as a controllable, but not yet high-yield, model system for basic plant EV biology and potential applications (e.g., nanocarriers (Yugay et al. 2023)). However, optimization of exogenous culture conditions, including growth regulator and ion concentrations, applies specifically to the in vitro system and cannot be directly extrapolated to cells in planta, which are influenced by endogenous gradients, tissue-specific properties and distinct epigenetic states. Within these limitations, suspension cell cultures remain particularly relevant for the study of EVs, as they allow for their reproducible production under defined and standardized conditions. This approach could be valuable for studying EV-mediated cell communication. Kocholatá et al. investigated EVs isolated from different *Nicotiana tabacum* tissues (leaves, apoplastic fluid, calli, and suspension cell cultures) and demonstrated that EVs can be reproducibly generated and purified from in vitro cell cultures, with morphological features comparable to those derived from whole tissues (Kocholatá et al. 2024). However, compositional variations in EVs indicate distinct cellular origins and functions (Kocholatá et al. 2024). A further limitation concerns the stability of plant cell cultures during prolonged propagation. Repeated subculturing may promote somaclonal variation and genetic or epigenetic drift (Miguel and Marum 2011). These changes could modify cellular metabolism and secretion pathways, potentially producing temporal shifts in EV abundance and cargo composition; however, their direct effects on plant EV profiles remain largely unexplored. Future studies should therefore report cell-line origin, culture age, subculture number and growth-regulator regime. The use of master cell banks, restricted subculture intervals and longitudinal molecular profiling would help monitor production stability and enable periodic comparison with EVs obtained from the corresponding native plant organs. The main advantages and limitations of plant cell cultures as platforms for EV production are summarized in Fig. 2.

Together, these findings indicate that in vitro plant cell cultures provide a unique opportunity to merge plant biotechnology and biomedicine, allowing for the scalable generation of EVs with defined features. In particular, defined culture conditions may facilitate reproducible EV production, controlled modulation of vesicle cargo and reduced variability compared with preparations obtained through whole-tissue disruption. Compared with mammalian cell cultures, plant cell cultures can generally be maintained in simpler, animal-component-free media without serum, potentially reducing upstream cultivation costs, ethical concerns and the risk of contamination by mammalian pathogens (Lian et al. 2022; Kırbaş et al. 2025). They also provide renewable biomass and are compatible with contained bioreactor production. However, these advantages do not eliminate the need for stringent downstream purification, quality control and species-specific process optimization. Translation of plant cell cultures to larger production scales also requires control of bioprocess parameters that may affect cell viability, EV yield, and cargo composition. These parameters include hydrodynamic shear stress, mixing, aeration and oxygen transfer, nutrient-feeding strategies, pH, temperature, and biomass density. Stirred systems may expose cells to damaging shear forces, whereas airlift and temporary immersion bioreactor systems may provide gentler mass transfer and permit controlled, non-destructive collection of EVs from the extracellular medium. Nevertheless, bioreactor configuration and operating conditions require species-specific optimization and systematic monitoring to ensure batch-to-batch consistency (Shu et al. 2025a, b).

## Plant extracellular vesicles: isolation, characterization, and molecular cargo

### Isolation and characterization strategies

The separation and purification of EVs from plants (PDEVs) remains one of the major obstacles in the field. In fact, it is considerably more difficult than purifying EVs from mammalian biological fluids or cells, due to the higher complexity of plant tissues (Duan et al. 2026).

Furthermore, pigments, phenolic compounds, starch, proteins, metabolites, and secondary metabolites are abundant in plant extracts and can coprecipitate with vesicles during the isolation process, interfering with EV purity and influencing their biochemical and functional analyses (Lian et al. 2022). In addition, PDEVs display diverse morphology and size distributions, and these variations challenge the identification and analysis of PDEVs using traditional methods (Li, Wang et al. 2025a, b). In recent years, their isolation has been achieved from various sources, including leaves, roots, fruits, and seeds. These materials can be blended to obtain a mixture that is then used for the isolation of PDEVs (Urzì et al. 2023). An alternative approach could be to isolate EVs released by roots. De Palma et al. isolated EVs from the roots of tomato plants grown in a hydroponic system. The EVs released were found to carry homologs of proteins typically associated with mammalian EVs, as well as with plant-derived apoplastic vesicles (De Palma et al. 2020). Several studies have also demonstrated the biological activity of PDEVs. For example, EVs isolated from strawberries and lemons showed antioxidant activity in vitro on adipose derived-mesenchymal stem cells (ADMSCs) (Perut et al. 2021). Similarly, D’Adduzio et al. isolated PDEVs from rosemary leaves and coffee grounds. Both matrices can be used as sources for EVs isolation, although rosemary leaves allowed the recovery of higher-quality EVs compared to coffee grounds. Nevertheless, EVs derived from both sources exerted positive effects on myotubes and fibroblasts (d’Adduzio et al. 2026). Although several research laboratories are currently isolating EVs from plants, the isolation method itself still remains a crucial factor. Depending on the source type, sample preparation may involve either pressing the fruit or plant tissue or collecting the apoplastic washing fluid (AWF). Huang et al. developed a protocol for AWF extraction from *Arabidopsis* leaves based on vacuum infiltration–centrifugation. This approach enabled the isolation of a purer AWF compared with the conventional method, which relies on vacuum aspiration of plant tissues followed by centrifugation (Huang et al. 2021).

Once the plant-derived starting material, such as tissue homogenate, expressed juice, or AWF, has been collected and pre-cleared, the PDEVs must be isolated. Methods for isolating EVs include ultracentrifugation, size-exclusion chromatography (SEC), ultrafiltration, precipitation, and immunoaffinity capture (Lian et al. 2022). In brief, these techniques exploit the physical properties of EVs, including size, density charge, solubility, and surface-exposed proteins, to separate them from biological contaminants. Among these approaches, differential ultracentrifugation (DUC) is currently the most commonly used technique for their isolation. It involves a series of differential centrifugation steps performed at 4 °C, followed by a final ultracentrifugation step. After ultracentrifugation, the EV pellet can be resuspended in an appropriate buffer (López de las Hazas et al. 2023). However, one limitation of DUC is that the high centrifugation speeds may cause the co-sedimentation of protein complexes and aggregates. In light of this limitation, several researchers have combined DUC with purification techniques to improve EV purity (Lian et al. 2022). One example is the combination of DUC with size-exclusion chromatography (SEC) (López de las Hazas et al. 2023; Steć et al. 2025).

SEC is a size-based separation method in which the mobile phase flows and is rinsed through a stationary phase, usually made of porous gel polymers. Because of its high purity and recovery rates, as well as the comparatively small sample amount needed, SEC has been used extensively for exosome isolation in recent years. Additionally, this method keeps exosomes and their molecular cargo structurally intact, allowing their further analysis and use (Thomas et al. 2024). Conversely, a comparison between ultracentrifugation and SEC revealed that EVs isolated using SEC exhibited enhanced functional performance, as this method better preserved their morphological features and structural integrity (Garzo et al. 2023). EVs with greater purity can also be separated by combining DUC with density-gradient ultracentrifugation (DGUC). The technique has been successfully used to separate plant EVs from different plants, including *Citrus limon*, *Portulaca oleracea*, and *Centella asiatica*, enabling the study of their biochemical composition and potential applications. This technique isolates PDEVs by exploiting their distinctive buoyant density (usually 1.10–1.18 g/mL) using media containing sucrose or iodixanol. Nevertheless, this approach can be quite time-consuming and decrease the final yield (Xiao et al. 2025). Another method is PEG (polyethylene glycol) precipitation, which induces vesicle precipitation by reducing their solubility due to polymer-induced crowding effects, favoring aggregation and sedimentation. However, this method can also lead to coprecipitation of non-vesicular components. In comparison with ultracentrifugation, PEG precipitation provided a high yield of vesicles, but showed traces of coprecipitated macromolecules, which is a known disadvantage of polymer-based methods (Kollannur and Gayathri 2026). These contaminants may contain soluble non-vesicular proteins, plant-derived polysaccharides, and aggregates of phenolic compounds, which co-sediment non-specifically with vesicles. Despite ultracentrifugation being the gold standard procedure for the isolation of PDEVs, PEG-based precipitation can be used as an alternative method due to its low cost, scalability, and accessibility, particularly in laboratories that lack ultracentrifugation equipment (Kollannur and Gayathri 2026). Alternatively, the extract may be purified using the Exosome Ultrafiltration System (EXODUS), which employs negative-pressure oscillation membrane vibration driven by coupled harmonic oscillators to facilitate efficient exosome separation (Ma et al. 2026a, b). This approach enables the recovery of highly purified EVs. Comparative studies have shown enhanced enrichment of exosomal mammalian EV markers, including Alix, CD63, and CD9, as well as reduced contamination by non-vesicular proteins (Chen et al. 2021). Tangential flow filtration (TFF) is increasingly used for EV isolation because it enables scalable and pharmaceutical-grade production. EVs isolated from *Citrus sinensis* juice by TFF showed physicochemical and morphological properties comparable to those obtained by ultracentrifugation, while exhibiting lower protein contamination due to the removal of non-vesicular proteins during diafiltration (Gai et al. 2026). Thus, TFF can be considered as an effective alternative to conventional ultracentrifugation for large-scale EV isolation (Kang et al. 2026).

Another promising isolation method is based on hydrophobic interaction chromatography (HIC) using a polyester (PET)-channeled polymer fiber (C-CP) stationary phase. The intrinsic hydrophobicity of the PET fibers, combined with a mild HIC solvent system, enables efficient and vesicle-preserving EV capture and release through a high-to-low salt concentration gradient (Jackson et al. 2023). Initially implemented in an HPLC format with online absorbance-based EV detection and quantification, the method was later adapted into a clinically practical solid-phase extraction (SPE) spin-tip format. The platform enables rapid (< 15 min) isolation of highly concentrated, high-purity, and bioactive EVs from diverse biofluids, with performance comparable or superior to established EV isolation methods (Jackson et al. 2023). Beyond these established approaches, several emerging technologies are being explored to further improve EV isolation. Microfluidic platforms have shown promising results by enabling label-free, high-throughput, and reproducible EV separation while preserving vesicle integrity (Abreu et al. 2022; Wang et al. 2024a, b). Acoustofluidic systems exploit acoustic waves to isolate EVs in a continuous and non-invasive manner, minimizing vesicle aggregation and preserving bioactivity (Wang et al. 2024a, b; Hu and Gao 2025). Deterministic lateral displacement (DLD) devices provide high-resolution size-based separation without the need for ultracentrifugation and may represent an attractive alternative for future EV purification workflows (Zhao et al. 2023a, b; Wang et al. 2024a, b; Rawat et al. 2025). Although these emerging technologies have demonstrated considerable potential for EV isolation, further validation and optimization will be required to establish their applicability to plant-derived extracellular vesicles, as the heterogeneous composition of plant-derived matrices and vesicle preparations poses unique methodological challenges.

The main isolation methods currently used for PDEVs, together with their principles, advantages, limitations, and representative references, are summarized in Table 1.

**Table 1:** Overview of the principal methods used for the isolation of plant-derived extracellular vesicles (PDEVs)

Isolation ,ethod	Principle	Advantages	Limitations	Representative references
Differential ultracentrifugation (DUC)	Sequential centrifugation steps separate particles according to their size and density	Widely used; relatively inexpensive; established protocols	Co-isolation of proteins and aggregates; potential vesicle damage; time-consuming	López de las Hazas et al. (2023), Xiao et al. (2025)
Density-gradient ultracentrifugation (DGUC)	Separation based on buoyant density using sucrose or iodixanol gradients	Higher purity and improved separation of EV subpopulations	Labor-intensive; low throughput; reduced yield	Xiao et al. (2025)
Size-exclusion chromatography (SEC)	Separation according to particle size through porous stationary matrices	High purity; preserves EV morphology and cargo integrity; reproducible	Sample dilution; incomplete removal of similarly sized contaminants	Garzo et al. (2023), Thomas et al. (2024)
Polyethylene glycol (PEG) precipitation	Polymer-induced crowding reduces EV solubility, promoting aggregation and sedimentation	Simple, inexpensive, scalable, high yield	Coprecipitation of proteins, polysaccharides, and phenolic compounds	Kocholata et al. (2022), Kollannur and Gayatrhi (2026)
Exosome ultrafiltration system (EXODUS)	Negative-pressure oscillation membrane vibration for EVs purification	Rapid isolation of highly purified EVs; reduced protein contamination	Limited application in plant EV studies; specialized equipment required	Chen et al. (2021); Ma et al. (2026a, b)
Tangential flow filtration (TFF)	Cross-flow filtration allows continuous EV concentration and purification	Scalable; suitable for pharmaceutical-grade production; lower protein contamination	Requires dedicated instrumentation and process optimization	Kang et al. (2026)
Hydrophobic interaction chromatography (HIC)	EVs interact with hydrophobic polyester fibers and are eluted using a salt gradient	Rapid isolation; high purity; preserves EV bioactivity; suitable for diverse plant sources	Limited validation compared with conventional methods	Bruce et al. (2019); Jackson et al. (2023)

The characterization of PDEVs is essential to verify successful isolation and assess sample purity through complementary physicochemical and structural parameters, including particle size, zeta potential and morphology (Alzahrani et al. 2023).

Usually, the morphological characterization of EVs is carried out using transmission electron microscopy (TEM) and cryo-electron microscopy (cryo-TEM), which facilitates the visualization of the characteristic membrane-enclosed nanovesicles and assessment of their structural integrity (Chen and Cai 2023; Ahmad et al. 2024). Particle size distribution and concentration are determined by nanoparticle tracking analysis (NTA) (Huang et al. 2025a, b). Furthermore, dynamic light scattering (DLS) and tunable resistive pulse sensing (TRPS) could provide complementary information on vesicle heterogeneity and size profiles (Szatanek et al. 2017; Misra et al. 2024). In addition, Zeta potential measurements of surface charge are commonly used to evaluate colloidal stability and forecast vesicle behavior in suspension (Midekessa et al. 2020).

Colorimetric tests including the bicinchoninic acid (BCA) assay are frequently used to measure total protein concentration (Cao et al. 2019). While for mammalian EVs, markers have been well established (such as CD9, CD63, and CD81), no uniform markers for plant EVs currently exist. However, several proteins have been identified as candidate markers for specific PDEV subpopulations. TETRASPANIN8 (TET8) has been proposed as a marker of exosome-like vesicles originating from multivesicular bodies in *Arabidopsis* (Cai et al. 2021). Thus, the most frequently detected proteins are TET8, PEN3, HSP70, HSP90, and PATL1, which are recognized indicators of plant EVs (Ekanayake et al. 2024). However, none of these proteins can currently be considered a universal plant EV marker. Their abundance may vary according to plant species, tissue, physiological state and EV subpopulation. Moreover, conserved proteins such as HSP70 and HSP90 are not sufficiently EV specific when used alone. Antibodies developed against mammalian EV markers cannot be assumed to recognize homologous plant proteins specifically; therefore, any apparent reactivity should be confirmed using orthogonal methods and appropriate controls. Plant-specific characterization guidelines should combine multiple candidate positive markers with negative markers for intracellular and organelle-derived contaminants, in line with the general principles of MISEV (Théry et al. 2018; Pinedo et al. 2021; Rodríguez de Lope et al. 2024).

#### Distinguishing plant extracellular vesicles from plant-derived nanovesicles

One of the major unresolved issues in the plant vesicle field concerns the precise nature of the particles recovered from different plant sources and isolation workflows. Although terms such as plant extracellular vesicles (PDEVs), plant-derived extracellular vesicles (PDEVs), plant-derived nanovesicles (PDNVs), and plant-derived exosomes are frequently used interchangeably, these preparations may originate from fundamentally different biological processes and therefore should not automatically be considered equivalent entities (Ambrosone et al. 2023; Mizenko et al. 2024; Zhang et al. 2026). PDEVs are released through regulated secretion pathways and accumulate in the apoplastic space. Current evidence suggests that their biogenesis involves endosomal trafficking pathways, including multivesicular bodies and ESCRT-associated mechanisms, resulting in the secretion of membrane-bound vesicles carrying proteins, lipids, and regulatory RNAs involved in intercellular communication (Thieron et al. 2024; Mosesso and Isono 2025). In contrast, PDNVs are generally obtained through mechanical disruption procedures such as homogenization, grinding, blending or extrusion of plant tissues (Alfieri et al. 2021; Woith et al. 2021). These approaches release not only extracellular vesicles but also intracellular vesicles, membrane fragments, organelle-derived particles, and soluble cellular components, generating highly heterogeneous preparations (Alfieri et al. 2021; Woith et al. 2021). Beyond their different biological origins, secreted EVs and tissue-disruption nanovesicles may differ in membrane topology and proteomic composition. Physiologically secreted EVs are expected to preserve the membrane orientation established during regulated biogenesis. Conversely, mechanical homogenization releases membranes from multiple intracellular compartments and may therefore generate particles with heterogeneous membrane orientations. However, direct comparative evidence on membrane topology in plant vesicular preparations remains limited. At the proteomic level, enrichment of candidate EV-associated proteins, such as TET8, PEN3 and PATL1, together with limited representation of chloroplast, mitochondrial or endoplasmic-reticulum proteins, may support—but does not conclusively demonstrate—an extracellular origin (Liu et al. 2020a, b; Kocholata et al. 2022; Bifolco et al. 2026).

Consequently, vesicles recovered from apoplastic fluids or extracellular culture media are more likely to represent physiologically secreted populations involved in cell-to-cell communication and environmental responses (De Palma et al. 2020; Liu et al. 2020a, b; Cho et al. 2021; Schatz et al. 2021; Nguyen et al. 2023; Yugay et al. 2023; Zhao et al. 2023a, b; Sánchez-López et al. 2024). In addition, non-vesicular contaminants such as soluble proteins, polysaccharides, pigments and cell wall-derived materials may co-purify with vesicular fractions and contribute to biological activities that are subsequently attributed to vesicles themselves (Minjares-Fuentes et al. 2017; Phillips et al. 2021; Thompson and Papoutsakis 2023). As a result, differences in isolation strategy can significantly influence both molecular characterization and functional outcomes.

Several studies have reported significant differences between extracellular vesicles obtained from apoplastic sources and nanovesicles derived from homogenized tissues. For example, vesicles isolated through non-destructive collection from apoplastic fluid or conditioned cell culture medium generally display smaller particle sizes, more consistent molecular profiles, and enrichment in EV-associated proteins, whereas tissue-derived preparations often exhibit broader size distributions, greater molecular complexity, and increased RNA content, likely reflecting the contribution of intracellular materials released during tissue disruption (Liu et al. 2020a, b; Cho et al. 2021; Kocholata et al. 2022; Zhao et al. 2023a, b).

The issue is particularly relevant when considering plant cell cultures as experimental and biotechnological platforms. Unlike tissue-derived preparations, suspension cultures allow the collection of vesicles directly from conditioned culture media, reducing interference from intracellular contents released during tissue processing and facilitating the implementation of standardized purification workflows (Cho et al. 2021; Schatz et al. 2021; Kocholata et al. 2022). This characteristic represents a major advantage for both controlled mechanistic studies in cultured cells and future biotechnological applications, where reproducibility, purity, and batch-to-batch consistency are essential requirements. However, findings obtained from suspension cell cultures cannot be directly extrapolated to the physiology of intact plants.

Taken together, these observations indicate that extracellular vesicles secreted by living plant cells and nanovesicle preparations obtained through tissue disruption should not be considered equivalent entities. Future studies should clearly report the biological source, collection strategy, and isolation workflow used, as these factors can profoundly influence vesicle composition, purity, yield, and biological activity. Despite significant advances in isolation technologies, the assessment of vesicle purity remains a major challenge in the plant EV field. Most studies rely on particle size distribution, morphology, and particle concentration, whereas systematic evaluation of co-isolated contaminants is often limited. As a consequence, preparations obtained using different isolation workflows may contain substantially different proportions of vesicular and non-vesicular components. The implementation of standardized quality control criteria, including particle-to-protein ratios, complementary characterization techniques, and the assessment of both positive and negative markers, would improve comparability among studies and facilitate the development of reproducible isolation procedures. Furthermore, detailed reporting of source material, isolation workflow, recovery yield, and purity metrics should become standard practice in future investigations (Théry et al. 2018; Kim and Kim 2022; Maricchiolo et al. 2025).

### Molecular cargo of plant EVs

PDEVs have been found to contain four main components: lipids, proteins, nucleic acids, and secondary metabolites. Each of these components may be essential to the stability and functionality of PDEVs (Lian et al. 2022). In this context, proteomic, transcriptomic, lipidomic, and metabolomic analyses are essential for the molecular characterization of PDEVs, enabling the identification of vesicle-associated cargo and potential molecular markers (Shaba et al. 2022).

Proteins found in PDEVs are thought to regulate important physiological processes in plants. For example, membrane proteins might help mammalian cells absorb and internalize PDEVs (Lian et al. 2022).

Proteomic analysis revealed that PDEVs contain proteins derived from various cellular compartments, suggesting their complex biogenesis. Interestingly, some proteins generally associated with mammalian EVs, such as HSP70, actin, and elongation factor 1-alpha (EF1α), were consistently identified across different PDEV populations, underlining their potential use as candidate markers. However, further validation is required, for which the lack of plant-specific antibodies remains a major challenge for PDEV standardization (Wang et al. 2025a, b). For example, proteomic analysis of *Arabidopsis* EVs identified proteins involved in vesicular trafficking such as syntaxins (coded by the genes *PEN1*/*SYP121*, *SYP122*, *SYP132*, and *SYP71*), RAB GTPases, and PATELLIN proteins, as well as many stress- and immunity-related proteins. They included RIN4, a key regulator of plant defense, and ROS-related proteins, including PLDα, PLDδ, ANNEXIN1, ascorbate peroxidase 1, and glutathione S-transferase PHI2 (Rutter and Innes 2017). This supports the function of EVs in immune responses and ROS signaling. Proteomic analysis of nanovesicles isolated from *Murraya koenigii* also revealed conserved proteins commonly reported in plant EVs, such as HSP70, HSP90, glutathione S-transferases, enolase, and hydrolases (Kollannur and Gayathri 2026). The enrichment of these functionally important proteins further supports the idea that protein cargo is selectively packaged into plant-derived EVs.

Several studies have reported the presence of small RNAs (sRNAs), including microRNAs (miRNAs), within plant-derived extracellular vesicles (PDEVs). These molecules play crucial roles in the regulation of plant development and immunity, particularly during responses to biotic and abiotic stress. Although the possibility of cross-kingdom miRNA-mediated regulation remains controversial, specific miRNAs have been consistently identified in PDEVs (Betti et al. 2021). For instance, *Fragaria*-derived exosome-like nanovesicles were found to be enriched in miR166g, a miRNA known to regulate developmental processes in plants. In *Arabidopsis*, overexpression of miR166g results in defects in shoot apical meristem development, stem vascularization, and leaf morphogenesis, highlighting its important regulatory functions (Perut et al. 2021). These findings suggest that PDEVs may serve as vehicles for the transport of biologically active regulatory RNAs. Interestingly, RNA profiling of *Citrus limon* EVs revealed the presence of small RNAs with a different size distribution (Baldini et al. 2018). The selective enrichment of 23-nucleotide short RNAs suggests the existence of active RNA sorting mechanisms during vesicle synthesis, although no specific miRNAs could be unequivocally identified (Baldini et al. 2018).

In contrast to mammalian EVs, PDEVs have a distinct lipid profile, with phosphatidic acid (PA), phosphatidylethanolamine (PE), and phosphatidylcholine (PC) being among their major lipid components (Huang et al. 2025a, b). These lipid species are presumed to be involved in critical functions, including membrane organization and stability, modulation of interactions between vesicles and cells, cellular uptake, and the biological activity of PDEVs. For example, *Arabidopsis*’ EVs are enriched in sphingolipids, particularly glycosylinositol phosphorylceramides (GIPCs), which is one of the most characteristic lipid classes found. Enrichment of GIPCs and other complex sphingolipids, often with very-long-chain fatty acids, is thought to be involved in membrane organization and signaling functions (Liu et al. 2024a, b).

Phospholipids are a major component of PDEV membranes, and phosphatidylcholine (PC) and phosphatidic acid (PA) are among the most abundant species. Importantly, PA has attracted great interest for its putative role in membrane remodeling, vesicle trafficking, and interaction with target cells. Furthermore, sterols, including sitosterol and campesterol, are consistently found in PDEVs, confirming that plants tend to store sterol esters in lipid droplets rather than in membranes (Liu et al. 2020a, b). Interestingly, ginger-derived EVs showed a unique lipid profile, with higher abundance of fatty acyls and the presence of bioactive ginger components including gingerols and shogaols (Zhuang et al. 2015; Wang et al. 2025a, b). These compounds, which have been previously reported in ginger extracts, have been validated in the vesicular fraction and are associated with biological activity in vitro. The high diversity of lipid composition across PDEVs suggests that lipid cargo may play roles in membrane structure and vesicle stability, as well as in their biological functions, cellular uptake, and potential therapeutic effects (Wang et al. 2025a, b).

Moreover, PDEVs contain different metabolites based on the plant species and the specific portion of the plant from which they are isolated. Alkaloids, flavonoids, and phenolic compounds are examples of these metabolites. It has been reported that most of these metabolites are produced to enable the plant to defend itself against pathogens, as well as to mediate biological effects, such as anti-inflammatory activity, in other cells (Karabay et al. 2025). Metabolomic profiles may be influenced not only by plant species and tissue origin but also by culture conditions and production systems. In Pinellia ternata, non-targeted metabolomics revealed distinct profiles between EVs collected from temporary immersion bioreactor medium and PDNVs obtained from plant tissues, while showing high consistency among biological replicates. These findings indicate that controlled culture systems may improve cargo reproducibility, but may also generate vesicles with metabolic profiles that differ from those of native organs. Secondary metabolites associated with edible or medicinal plants may contribute to therapeutic activity; however, their localization within the vesicle lumen or membrane should be distinguished from co-isolated free compounds. In parallel, lipid composition can influence membrane organization, permeability and resistance to oxidation, although direct causal relationships between individual metabolites, vesicle stability, and therapeutic efficacy remain insufficiently defined (Woith et al. 2021; Lian et al. 2022; Shu et al. 2024). An example has been reported for PDEVs isolated from *Cannabis*. These vesicles were shown to reduce the viability of two hepatocellular carcinoma cell lines, an effect that is probably associated with their cannabidiol (CBD) content. PDEVs were isolated from two different cannabis chemotypes, and vesicles derived from one chemotype contained higher levels of CBD than those obtained from the other. Notably, the CBD-enriched PDEVs exhibited a greater ability to reduce cancer cell viability, suggesting that the anticancer activity of cannabis-derived EVs may be at least partially mediated by their CBD cargo (Tajik et al. 2022). Vitamin C (ascorbic acid), a natural antioxidant that protects plant cells against oxidative stress, is an essential nutrient in humans and acts as a cofactor in numerous enzymatic reactions. Notably, extracellular vesicles derived from *Fragaria* and *Citrus L* were identified as carriers of vitamin C and exhibited antioxidant activity, suggesting that vesicle-associated ascorbic acid may contribute to the biological effects of these nanovesicles (Baldini et al. 2018; Perut et al. 2021). Additionally, encapsulation within the vesicular structure protects vitamin C from degradation, thereby enhancing its stability and bioavailability compared with the free molecule (Baldini et al. 2018).

## Biological activities and therapeutic potential of plant-derived extracellular vesicles

PDEVs are increasingly recognized as bioactive vesicular entities with potential relevance in inter-kingdom communication, natural cargo delivery and biomedical applications. Their heterogeneous molecular composition, including proteins, lipids, metabolites and regulatory RNAs, may support combined biological effects across different cellular and tissue contexts. Experimental evidence indicates that PDEVs can modulate inflammatory and immune responses, contribute to intestinal homeostasis, promote regenerative processes, exert selective anticancer effects, and display neuroprotective activity in preclinical models. These effects have been associated with mechanisms such as regulation of oxidative stress, immune cell reprogramming, microbiota modulation, stimulation of cell proliferation and migration, induction of tumor cell death, and preservation of neuronal or vascular function. However, despite these promising findings, further studies are required to clarify vesicle biodistribution, uptake mechanisms, immune interactions, active cargo components and long-term safety. Functional studies should also exclude the contribution of co-isolated free metabolites, including phenolics, flavonoids, and polysaccharides. Biological activity should be compared among intact EV preparations, matched EV-depleted supernatants or non-vesicular fractions, and vesicles disrupted by detergent, sonication, or freeze–thaw cycles. Proteinase K treatment, performed with and without membrane permeabilization, may help distinguish effects mediated by surface-exposed proteins from those associated with protected intravesicular cargo. However, loss of activity after vesicle disruption should be interpreted together with purification controls and quantitative cargo analyses, as disruption may also alter the stability of bioactive molecules (Théry et al. 2018; Pinedo et al. 2021; Lian et al. 2022). A summary of the main biological activities, proposed mechanisms, and experimental evidence supporting the biomedical potential of PDEVs is provided in Table 2.

**Table 2:** Biological activities, therapeutic applications, proposed mechanisms of action, and level of experimental evidence for plant-derived extracellular vesicles (PDEVs)

Application area	Disease/model	Plant source	Main mechanism of action	Biological target	Observed effect	Evidence Level	Ref
Inflammation/metabolism	Obesity	Various plants	microRNA-mediated regulation of macrophage metabolism	Macrophages, adipocytes	Improved metabolic balance, obesity prevention	In vivo	Bian et al. (2023)
Intestinal inflammation/drug delivery	DSS-induced colitis	Grapefruit	Uptake by intestinal macrophages, HO-1 upregulation, and inhibition of IL-1β and TNF-α	Intestinal macrophages	Reduced intestinal inflammation and improved methotrexate efficacy	In vivo	Wang et al. (2014)
Intestinal inflammation	Colitis	Ginger	Bidirectional cytokine modulation and epithelial support	Immune and epithelial cells	Reduced inflammation, enhanced repair	In vivo	Zhang et al. (2016)
Neuroinflammation	Cerebral ischemia–reperfusion injury	Panax notoginseng	Promotion of anti-inflammatory microglial polarization (lipid mediated)	Microglia	Reduced ischemic damage	In vivo	Li et al. (2023)
Intestinal inflammation	Colitis	*Portulaca oleracea*	Cytokine modulation and microbiota regulation	Immune cells, gut microbiota	Amelioration of colitis symptoms	In vivo	Zhu et al. (2023)
Oxidative stress	Oxidative damage	Ginger, lemon	Activation of antioxidant defense pathways	Multiple cell types	Protection from oxidative stress	In vivo	Urzì et al. (2023)
Microbiota regulation	Dysbiosis/colitis	Turmeric	Restoration of microbial diversity and abundance	Gut microbiota	Improved intestinal homeostasis	In vivo	Gao et al. (2022)
Microbiota regulation	Intestinal homeostasis	Buckwheat	Regulation of microbial metabolic genes	*Escherichia coli*, *Lactobacillus rhamnosus*	Enhanced microbial diversity	In vitro/in vivo	Liu et al. (2022)
Intestinal inflammation	Colitis	Garlic	Inhibition of inflammatory signaling and promotion of anti-inflammatory bacteria	Immune cells, gut microbiota	Reduced inflammation and disease severity	In vivo	Wang et al. (2024a, b)
Skin regeneration	Wound healing	Ginseng	Activation of proliferative and angiogenic signaling pathways	Keratinocytes, endothelial cells	Accelerated wound healing	In vivo	Yang et al. (2023)
Bone regeneration	Osteogenic differentiation	Rhizoma Drynariae	Upregulation of osteogenic regulators	Bone marrow mesenchymal stem cells	Enhanced osteogenesis	In vitro/In vivo	Zhao et al. (2024)
Muscle regeneration	Muscle atrophy	Gouqi	Activation of energy metabolism pathways	Skeletal muscle fibers	Improved muscle mass and strength	In vivo	Zhou et al. (2024)
Skin regeneration	Wound healing	Wheat, grapefruit	Stimulation of cell proliferation, migration, and extracellular matrix production	Keratinocytes, fibroblasts, endothelial cells	Improved wound closure and angiogenesis	In vitro / In vivo	Şahin et al. (2019); Savcı et al. (2021)
Oncology	Solid tumors (various models)	Multiple plant sources	Regulation of proliferation, apoptosis, metabolism, and tumor microenvironment	Cancer cells, tumor microenvironment	Reduced tumor growth and invasiveness	Preclinical	Kim et al. (2020); Chen et al. (2022); Chen et al. (2023); Zhao et al. (2023a, b)
Oncology	Solid tumors	Lemon and other citrus plants	Activation of apoptosis-inducing ligand signaling pathways	Cancer cells	Selective induction of cancer cell apoptosis	In vivo	Koornstra et al. (2003); Ishibashi and Ohtsuki (2008); Stanly et al. (2020); Zhou et al. (2022)
Oncology	Breast cancer	*Brucea javanica*	Delivery of regulatory microRNAs targeting growth and survival pathways	Cancer cells, endothelial cells	Suppressed tumor growth and angiogenesis	In vivo	Yan et al. (2024)
Oncology (Combination Therapy)	Oral squamous cell carcinoma	Bitter melon	Synergistic enhancement of chemotherapy efficacy	Cancer cells	Increased cytotoxicity and tumor suppression	In vitro/In vivo	Yang et al. (2021)
Neuroprotection	Parkinson’s disease (animal model)	*Pueraria lobata*	Enhancement of mitophagy and restoration of mitochondrial energy metabolism after blood–brain barrier crossing	Neurons, mitochondria	Improved motor performance and neuroprotection	In vivo	Xu et al. (2024)
Neuroprotection	Ischemic brain injury	*Momordica charantia*	Protection of blood–brain barrier integrity and inhibition of apoptosis via AKT and glycogen synthase kinase 3 beta signaling	Neurons, endothelial cells	Reduced infarct volume and brain damage	In vivo	Cai et al. (2022); Ye et al. (2024)
Neuroinflammation	Alcohol-induced brain inflammation	Oat	Regulation of microglial signaling complexes involved in innate immune activation	Microglial cells	Reduced neuroinflammation and improved cognitive function	In vivo	Xu et al. (2022)
Neuroinflammation	Neuroinflammatory models	*Allium tuberosum*	Reduction of inflammatory mediators and oxidative stress	Neural and immune cells	Attenuation of neuroinflammatory responses	In vitro / In vivo	Ishida et al. (2023)

### Engineering strategies to tailor plant-derived EVs

**Fig. 3:**
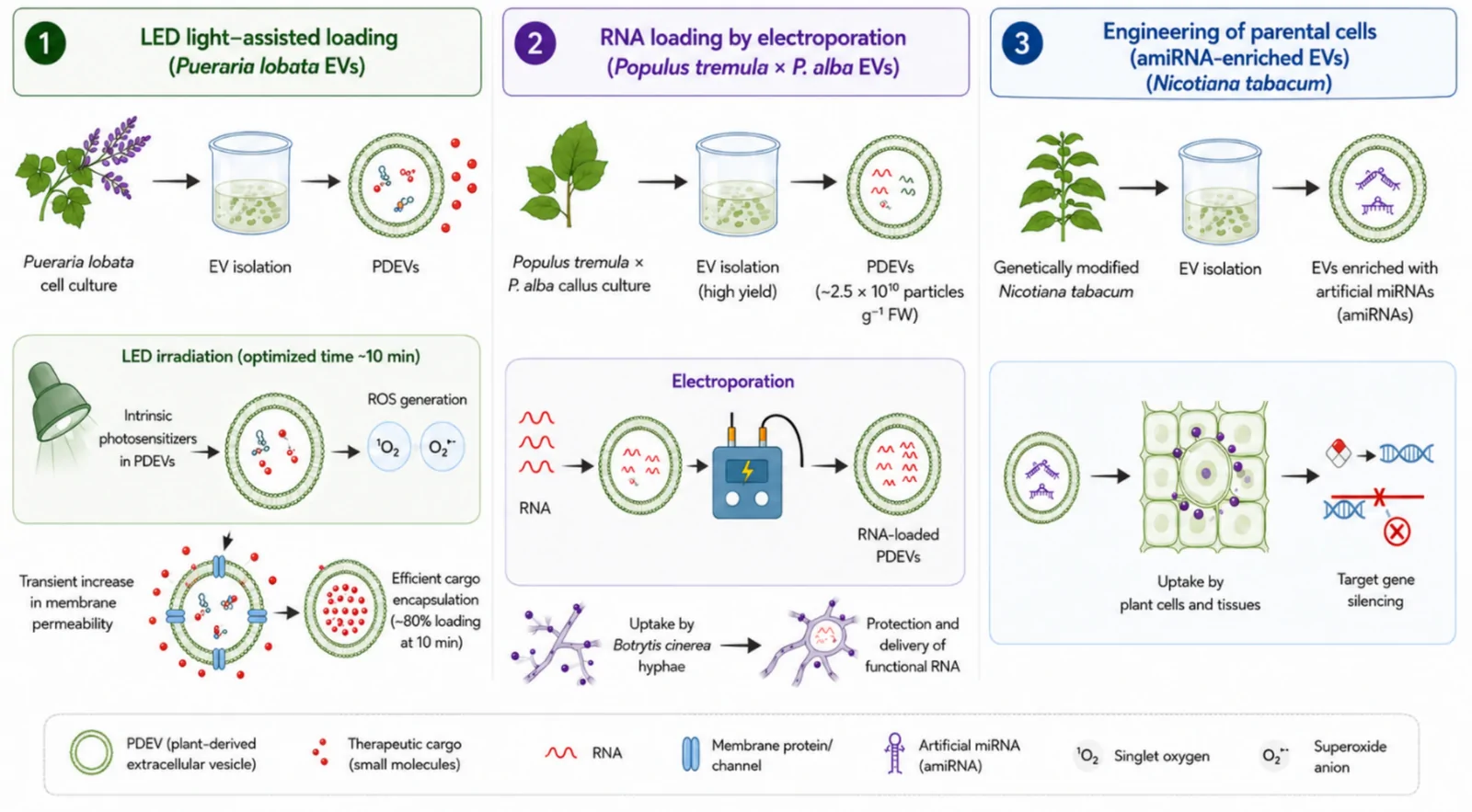
Engineering strategies to tailor plant-derived extracellular vesicles (PDEVs). Three complementary approaches are currently being explored to generate PDEVs with defined molecular cargo and biological functions. (1) LED-assisted loading transiently increases membrane permeability through reactive oxygen species (ROS) generation, facilitating efficient cargo encapsulation while preserving vesicle integrity under optimized irradiation conditions. (2) Electroporation enables the loading of RNA into PDEVs isolated from *Populus tremula* × *P. alba* callus cultures, allowing the delivery of functional RNA following uptake by *Botrytis cinerea*. (3) Genetic engineering of *Nicotiana tabacum* enables the production of PDEVs enriched with artificial microRNAs (amiRNAs), which are internalized by recipient plant cells and mediate sequence-specific gene silencing. Together, these approaches illustrate complementary strategies for engineering plant-derived extracellular vesicles with tailored cargo and biological functions

Besides their intrinsic biological activities, increasing efforts have focused on engineering plant-derived extracellular vesicles (PDEVs) for biotechnology and drug delivery applications. Different physical and chemical approaches have been developed to load PDEVs with RNA and other bioactive compounds, further expanding their potential as versatile delivery vehicles. In this context, Lu et al. investigated the use of LED light irradiation to enhance payload encapsulation into PDEVs while preserving their structural integrity under optimized irradiation conditions (Lu et al. 2025). The authors showed that LED irradiation exploits the intrinsic photosensitizing properties of PDEVs to generate reactive oxygen species (ROS), including singlet oxygen and superoxide anions. These ROS transiently increase membrane permeability, thereby facilitating controlled drug loading. An irradiation time of 10 min resulted in an optimal loading efficiency (~ 80%), whereas extending the exposure to 15 min reduced the loading efficiency by approximately 50%, most likely because excessive ROS generation caused membrane destabilization (Lu et al. 2025). These results indicate that *Pueraria lobata*-derived exosome-like nanovesicles are light-responsive nanocarriers that can balance the increased permeability induced by ROS with structural integrity. Therefore, light-mediated modulation enhances plant-derived EVs (PDEVs) as a scalable, non-invasive platform for photodynamic and precision drug delivery applications by modulating permeability changes without compromising structural integrity (Lu et al. 2025; Singh et al. 2025). Induced *Populus tremula* × *P. alba* callus cell lines further demonstrated the potential of plant in vitro cultures as scalable EV production platforms. EVs were isolated at high yields (approximately 2.5 × 10^10^ particles g^−1^ fresh weight) and successfully loaded with RNA by electroporation. The RNA-loaded vesicles were subsequently internalized by Botrytis cinerea hyphae, demonstrating their capacity to protect and deliver functional cargo (Rito et al. 2025). More recently, genetically modified Nicotiana tabacum plants were shown to produce EVs selectively enriched with artificial microRNAs (amiRNAs). These engineered EVs were efficiently internalized by recipient plant cells and tissues, where they induced sequence-specific silencing of the target gene, providing the first proof of concept that plant-derived EVs can be programmed for targeted RNA delivery. This study highlights the potential of engineering parental plant cells to generate EVs with predefined molecular cargo, opening new opportunities for plant biotechnology and RNA-based delivery applications (Shkryl et al. 2025). Collectively, these studies demonstrate that engineering approaches applied to plant cell cultures are transforming them from passive sources of extracellular vesicles into programmable production platforms capable of generating EVs with tailored cargos and defined biological functions. Representative strategies currently used to engineer PDEVs for cargo loading and functionalization are summarized in Fig. 3.

These engineering strategies involve different trade-offs in loading efficiency, vesicle integrity, potential cytotoxicity, and scalability (Table 3). LED-assisted loading can achieve high encapsulation efficiency under optimized conditions, but excessive irradiation may generate ROS and destabilize vesicle membranes (Lu et al. 2025). Electroporation is adaptable to different RNA cargos but may promote vesicle aggregation or membrane alteration and therefore requires method-specific optimization (Rito et al. 2025; Singh et al. 2025). Genetic modification of parental plant cells enables cargo enrichment during EV biogenesis without post-isolation membrane disruption, although the generation, validation and regulatory management of engineered cell lines may limit rapid implementation (Shkryl et al. 2025).

**Table 3:** Comparative features of principal PDEV engineering strategies

Engineering strategy	Loading efficiency	Vesicle integrity	Potential cytotoxicity	Scalability	Main limitation	References
LED-assisted loading	Approximately 80% under optimized irradiation conditions	Preserved at optimized exposure; reduced with excessive irradiation	Possible ROS-mediated oxidative damage	Potentially high	Requires optimization for each vesicle source and cargo	Lu et al. (2025)
Electroporation	Cargo and protocoldependent; not yet standardized for PDEVs	Possible membrane alteration or vesicle aggregation	Possible effects associated with membrane damage and residual free cargo	Moderate	Requires post-loading purification and protocol optimization	Rito et al. (2025); Singh et al. (2025)
Genetic modification of parental cells	Selective cargo enrichment, but quantitative efficiency remains incompletely defined	Preserved because loading occurs during EV biogenesis	No loading-related membrane damage; biological safety requires evaluation	Potentially high in stable cultures	Time required for cell-line generation, validation and regulatory control	Shkryl et al. (2025)

## Methodological gaps and unmet needs

Despite substantial progress in elucidating the biological functions and translational potential of PDEVs, key methodological challenges continue to impede their reproducible use and clinical translation. A major limitation in the field is the lack of standardized isolation and purification protocols, which complicates comparisons across studies and may lead to inconsistent interpretation of vesicle identity and function. Current workflows often adapt techniques such as differential ultracentrifugation and gradient purification from mammalian systems without critical validation in plant contexts, resulting in variable vesicle yield and co-isolation of contaminants (Kameli et al. 2021). Another major challenge concerns the lack of consensus regarding the classification of plant vesicular preparations. In particular, EVs secreted by living plant cells are frequently discussed together with nanovesicle populations obtained through tissue disruption, despite their distinct biological origins and compositional characteristics (Zhang et al. 2026). This ambiguity complicates comparisons among studies and may contribute to inconsistencies in reported biological activities. In addition, comprehensive characterization of PDEVs remains incomplete due to the absence of universally accepted markers and quality control criteria specific to plant vesicles. Unlike mammalian systems, where consensus markers are well established, PDEVs display diverse cargo profiles influenced by species, tissue source, and culture conditions, and these properties have not yet been captured by standardized characterization frameworks (Shu et al. 2025a, b). The limited understanding of vesicle biogenesis, cargo sorting mechanisms, and storage stability further constrains mechanistic insights and functional analyses. For instance, plant cell cultures exhibit different vesicle abundance and biogenesis signatures compared with intact plant tissues, underscoring the need for optimized culture and secretion models to investigate vesicle formation pathways (Zeng et al. 2024; Rito et al. 2025; Zhang et al. 2025). Functionally, most studies to date rely heavily on in vitro systems or acute in vivo models, with a paucity of rigorous investigations into long-term biodistribution, pharmacokinetics, and immune interactions following systemic administration. Detailed dose–response studies, mechanistic information on uptake pathways, and assessment of potential off-target effects remain largely unexplored, creating significant uncertainty for any therapeutic development (Liu et al. 2024a, b; Qiang et al. 2024; Zhang et al. 2025). Following systemic administration, PDEV development will require quantitative assessment of circulation time, tissue distribution, uptake by the mononuclear phagocyte system, and accumulation in clearance organs such as the liver and spleen (Liu et al. 2024a, b; Qiang et al. 2024). These parameters may vary according to vesicle size, lipid composition, plant source, administration route, and dose. Surface PEGylation could prolong circulation, whereas targeting ligands may improve tissue-specific uptake; however, both approaches may alter vesicle interactions, biological activity and immunogenicity and therefore require direct validation in plant-derived systems (Zhang et al. 2025). Preclinical evaluation should also include repeated-dose studies, complement activation, cytokine responses, antibody formation, hematological and biochemical parameters, organ histopathology, and long-term toxicity. Biodistribution should be confirmed using quantitative and orthogonal tracing methods to exclude signals derived from dissociated fluorescent labels. Collectively, these methodological gaps highlight the need for community-driven efforts toward guidelines for isolation, characterization, and functional validation of PDEVs, aligned with international standards such as those promoted by the International Society for Extracellular Vesicles (ISEV; https://www.isev.org/misev). Addressing these unmet needs will be essential for rigor, reproducibility, and eventual clinical translation of plant vesicle research.

## Future perspectives

Future progress in plant-derived extracellular vesicle research will require more standardized and mechanistic approaches. Plant-specific guidelines are needed to distinguish bona fide EVs secreted by living cells from heterogeneous nanovesicles generated by tissue disruption, and to harmonize isolation, characterization, and reporting criteria. A better understanding of EV biogenesis, including the contribution of multivesicular bodies, EXPOs, vacuolar pathways, and autophagy-related compartments, will also be essential. Robust plant EV markers and integrated imaging, proteomic, lipidomic and transcriptomic analyses will help improve vesicle classification and functional interpretation.

Plant cell cultures offer a valuable system to address these issues, as they allow vesicle collection from controlled extracellular environments and reduce variability associated with tissue disruption. Their scalability and compatibility with bioprocessing technologies may support reproducible EV production. However, these relatively homogeneous cultures lack the differentiated cell types, tissue organization and endogenous signaling gradients of intact plants; therefore, they cannot fully reproduce plant-level physiological or stress responses. Further translational studies should define vesicle cargo, dose normalization, uptake mechanisms, biodistribution, immune interactions and long-term safety, while establishing causal links between EV-associated molecules and biological effects.

## Conclusions

PDEVs are emerging as mediators of plant intercellular communication and as potential tools at the interface between plant biotechnology and biomedicine. They participate in defense, stress adaptation, and cross-organism interactions through the transfer of proteins, lipids, metabolites, and regulatory RNAs, while their biocompatibility and bioactive cargo support interest in therapeutic nanocarrier applications. However, progress is limited by unclear vesicle definitions, lack of standardized isolation methods, insufficient plant-specific markers, and limited quality-control criteria. Plant cell cultures may help overcome these limitations by providing controlled systems for studying EV biogenesis, function, and scalable production. However, their relative homogeneity and lack of tissue organization mean that they provide limited information about integrated physiological responses at the whole-plant level.

## Data Availability

No datasets were generated or analyzed during the current study.
